# NPR2 is involved in FSH-mediated mouse oocyte meiotic resumption

**DOI:** 10.1186/s13048-016-0218-y

**Published:** 2016-02-16

**Authors:** Lei Yang, Qiang Wei, Wei Li, Qihui Xi, Xiaoe Zhao, Baohua Ma

**Affiliations:** Key Laboratory of Animal Biotechnology of the Ministry of Agriculture, Northwest A&F University, Yangling, Shaanxi 712100 China; College of Veterinary Medicine, Northwest A&F University, Yangling, Shaanxi 712100 People’s Republic of China

**Keywords:** Meiotic resumption, Follicle-stimulating hormone, Epidermal growth factor receptor, Mitogen-activated protein kinase 3/1, Natriuretic peptide receptor 2

## Abstract

**Background:**

Previous studies have reported that follicle-stimulating hormone (FSH) is often added to culture media to induce oocyte meiotic resumption and maturation and to improve subsequent embryonic development during in vitro maturation (IVM). However, the underlying mechanisms remain unclear.

**Methods:**

Cumulus-oocyte complexes (COCs) were collected from ovaries 46–48 h after the female mice were intraperitoneally injected with 8 IU equine chorionic gonadotropin (eCG) and then the COCs were cultured in different medium. qRT-PCR analysis was used to assess mRNA expression of EGF-like factors and natriuretic peptide receptor 2 (NPR2). Western Blot analysis was used to assess phosphorylation of mitogen-activated protein kinase 3/1 (MAPK3/1). The oocytes were morphologically assessed for meiotic resumption.

**Results:**

FSH stimulated the expression of EGF-like factors, the activation of MAPK3/1, a decrease in NPR2 mRNA and oocyte meiotic resumption. Moreover, the FSH-induced decrease in NPR2 and oocyte meiotic resumption occurred via the MAPK3/1 singling pathway, which was activated by the epidermal growth factor receptor (EGFR) pathway.

**Conclusions:**

NPR2 is involved in FSH-mediated oocyte meiotic resumption, and this process is associated with the EGFR and MAPK3/1 signaling pathways.

## Background

During oocyte maturation, oocytes acquire the competence to progress through meiosis because they have assembled the necessary cell cycle machinery [1]. However, oocytes remain in this suspended state because signals from the granulosa cell compartment preclude the activation of this machinery that is required for entry into metaphase. The nature of these signals is now better understood; C-type natriuretic peptide (CNP) and its cognate receptor, the natriuretic peptide receptor 2 (NPR2) have been shown to promote cyclic guanosine monophosphate (cGMP) production in granulosa and cumulus cells [2]. cGMP diffuses into oocytes through gap junctions and inhibits the activity of phosphodiesterase 3A (PDE3A), resulting in the prevention of cyclic adenosine monophosphate (cAMP) degradation [3]. Elevated cAMP promotes the phosphorylation of cyclin-dependent kinase 1 (CDK1) and inactivates maturation-promoting factor (MPF) [4, 5] to maintain oocyte meiotic arrest.

When immature oocytes are isolated from follicles and cultured in vitro, the oocytes spontaneously resume meiosis because of their removal from the inhibitive environment of follicles [6, 7]. However, the subsequent developmental competence of oocytes matured in vitro is compromised compared with that of oocytes matured in vivo [8–10]. Gonadotrophin orchestrates the acquisition of oocyte competence, both in vivo and in vitro; therefore, in vitro culture systems of media were often supplemented with gonadotrophins, such as follicle-stimulating hormone (FSH), to induce cumulus cell expansion, nuclear maturation and cytoplasmic maturation and to improve embryonic development in many animals including the mouse [11–13], pig [14], cow [15], horse [16] and dog [17]. The molecular mechanisms by which gonadotrophins induce oocyte meiotic resumption is partly mediated by increasing the production of cAMP [18, 19] and subsequently activating mitogen-activated protein kinase 3/1 (MAPK3/1) in its surrounding cumulus granulosa cells [20–22]. Recent findings indicate that FSH induces the expression of epidermal growth factor (EGF)-like factors [23, 24] and activates the epidermal growth factor receptor (EGFR), which further activates MAPK3/1 [25]. In addition, recent studies reported that FSH decreased NPR2 expression in pig cumulus-oocyte complexes (COCs) [26] and the EGFR signaling pathway inhibited NPR2 expression via the MAPK3/1 signaling pathway in mouse COCs [27].

As mentioned above, EGFR, MAPK3/1 and NPR2 have been shown to participate in oocyte meiotic resumption; however, direct evidence indicating their relationships with FSH have not yet been reported. The present study aimed to determine the molecular mechanisms of FSH on oocyte meiotic resumption, especially the possible correlations among FSH, EGFR, MAPK3/1 and NPR2, and to elucidate the signal transduction pathways involved in this process.

## Methods

### Chemicals and mice

All chemicals were purchased from Sigma-Aldrich (St. Louis, MO, USA) unless otherwise stated. CNP, FSH and EGF were prepared as stock solutions in distilled PBS containing 0.1 % BSA. Stock solutions of the EGFR inhibitor AG1478 and the MAPK3/1 inhibitor U0126 (10 mM and 5 mM, respectively) were prepared in DMSO and stored at -20 °C. The final concentrations of EGF, AG1478 and U0126 for culture COCs were 100 ng/ml, 10 μM and 5 μM, respectively. Prior to use, they were diluted with culture medium, and the final concentration of DMSO was 0.1 %. Immature 21- to 35-day-old female mice (Kunming White outbred strain) were obtained from the Laboratory Animal Center of The Fourth Military Medical University. The mice were housed in an environment with controlled temperature and humidity, a 12 h light dark cycle, and ad libitum access to food and water. The present study was reviewed and approved by the Institutional Animal Care and Use Committee of College of Veterinary Medicine, Northwest A&F University.

### Oocyte collection, oocyte culture and oocyte assessment

In vitro oocyte maturation was performed as previously described with slight modifications [28]. COCs were collected from ovaries 46–48 h after the female mice were intraperitoneally injected with 8 IU equine chorionic gonadotropin (eCG). COCs were released from antral follicles by puncturing the follicles with a needle in culture medium. The culture medium used for this study was α-minimal essential medium (α-MEM; Invitrogen) containing 50 nM CNP, 0.23 mM sodium pyruvate, 2 mM glutamine, 3 mg/ml BSA, 75 IU/ml potassium penicillin G and 50 mg/ml streptomycin sulfate. LH, EGF, AG1478 and U0126 were added either alone or in combination at the start of culture. In each experiment, a group of 30 COCs were cultured in a 60 μl drop covered with paraffin oil in a 3.5-mm culture dish. All cultures were incubated at 37 °C in a humidified atmosphere of 5 % CO_2_ in air [29]. COCs were denuded mechanically by repeated pipetting to remove cumulus cells at the end of culture. The oocytes were then morphologically assessed for meiotic resumption.

### RNA isolation and qRT-PCR

The COCs of each treatment were pooled for RNA extraction. Total RNA was extracted using Trizol reagent (Invitrogen/Life Technologies, Carlsbad, CA). First-strand cDNA was synthesized according to the manufacturer’s instructions (PrimeScript® RT reagent Kit). qRT-PCR was performed using an ABI StepOnePlus Real-time Detection System (AB, CA, and USA) and SYBR Green qPCR SuperMix (Invitrogen, USA). Each experiment was repeated independently at least three times, and the fold change in the expression of each gene was analyzed using the 2-^ΔΔCT^ method. All of the primers were used as previously reported [2, 30], and the L19 ribosomal protein gene (RPL19) was used as the internal control.

### Western blot analysis

To detect phosphorylated MAPK3/1, approximately 120 COCs for each treatment were lysed using 120 μl 1 × SDS supplemented with 1 mM phenylmethylsulfonyl fluoride and 1 mM sodium orthovanadate for 20 min on ice, and then the samples were stored at -20 °C. Before electrophoresis, the samples were heated to 100 °C for 5 min, cooled on ice immediately, and then centrifuged at 12,000 g for 5 min. Each sample was separated by 12 % SDS-PAGE and electro-transferred onto a PVDF membrane. After incubation in blocking buffer for 1 h at 37 °C, the membrane was incubated overnight at 4 °C with anti-MAPK3/1, anti-pMAPK3/1 (Cell Signaling Technology; 1:1000 dilutions) or β-actin (1:1000; Beijing CWBIO Co., Ltd., Beijing, China) as a loading control. After washing, the membranes were incubated with secondary antibody conjugated to horseradish peroxidase at 37 °C for 30 min. Finally, immunoreactive bands were visualized using a Super Signal West Pico kit according to the manufacturer’s instructions.

### Statistical analyses

All experiments were repeated at least three times for each group, and the data are presented as the mean ± SEM. The data were analyzed by ANOVA, followed by Fisher’s least significant difference test and independent samples Student’s *t* test, with SPSS software, version 13.0 (SPSS, Chicago, IL, USA).

## Results

### FSH decreases NPR2 mRNA expression and promotes oocyte meiotic resumption

**Fig. 1:**
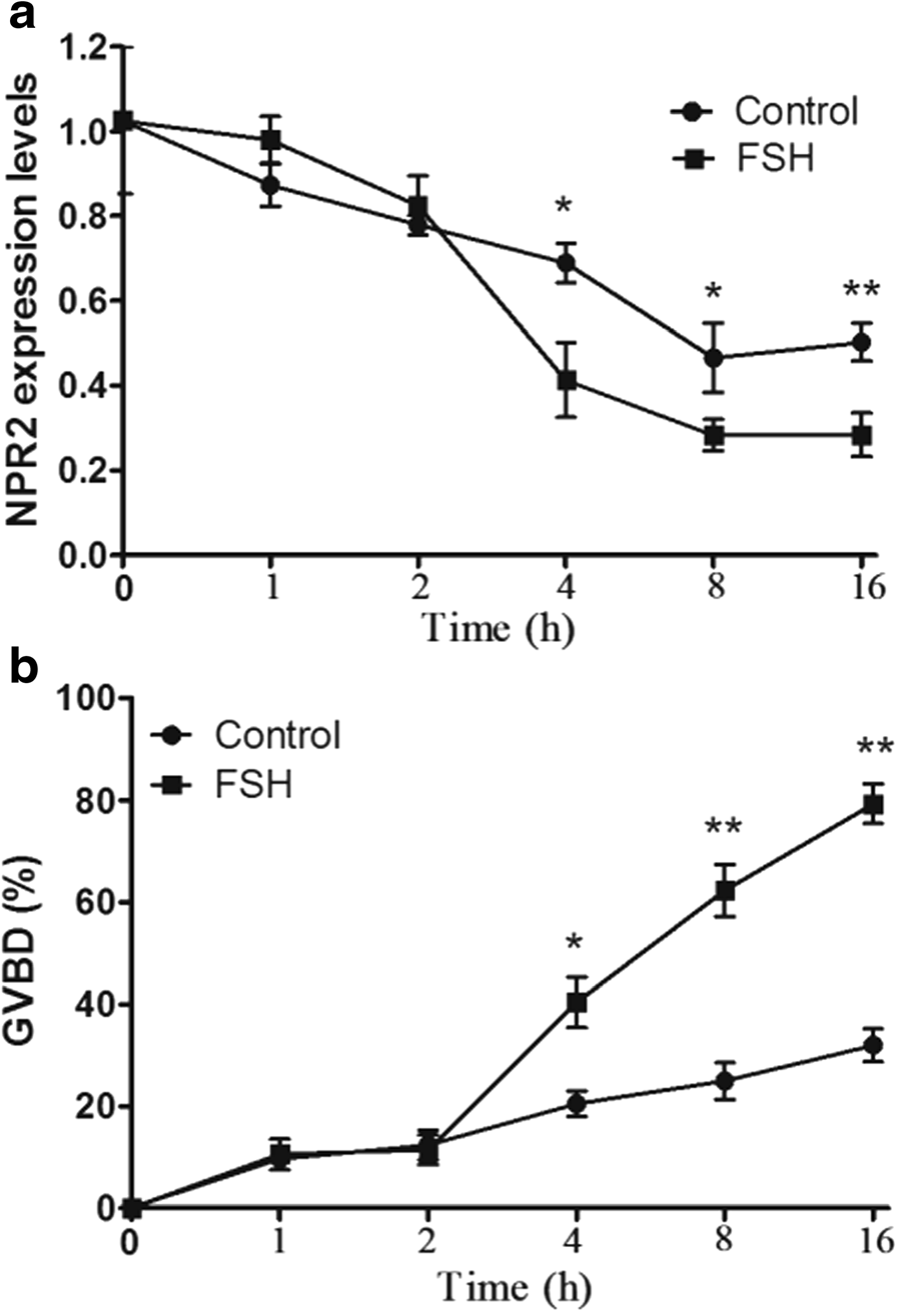
The effects of FSH on oocyte meiotic resumption and NPR2 mRNA expression in COCs. **a** Kinetics of oocyte meiotic resumption after FSH induction in COCs at different times. **b** Patterns of NPR2 mRNA expression after FSH induction in COCs at different times. The results are presented as the mean ± SEM and are representative of three independent experiments. *P < 0.05 and **P < 0.01 compared with each corresponding group in the control

To test the possibility that NPR2 signaling mediates FSH-induced oocyte meiotic resumption, we first examined the effects of FSH on oocyte meiotic resumption and NPR2 expression during oocyte maturation in vitro. The COCs were cultured with 2 IU/ml FSH for different lengths of time (0, 1, 2, 4, 8 and 16 h). The pattern of NPR2 mRNA expression levels during the culture in vitro was similar to the oocyte meiotic resumption rate (Fig. 1). We found that the expression of NPR2 in the FSH-stimulated COCs significantly decreased after 4 h of culture and remained at low levels until the end of the experiment at 16 h (Fig. 1a). The kinetics of germinal vesicle breakdown (GVBD) showed that, compared with control groups without FSH treatment, a significant increase in GVBD occurred 4 h after FSH stimulation (Fig. 1b) when the NPR2 mRNA expression levels were decreased to approximately 55 % of the baseline levels.

### FSH stimulates mRNA expression of EGF-like factors and phosphorylation of MAPK3/1

**Fig. 2:**
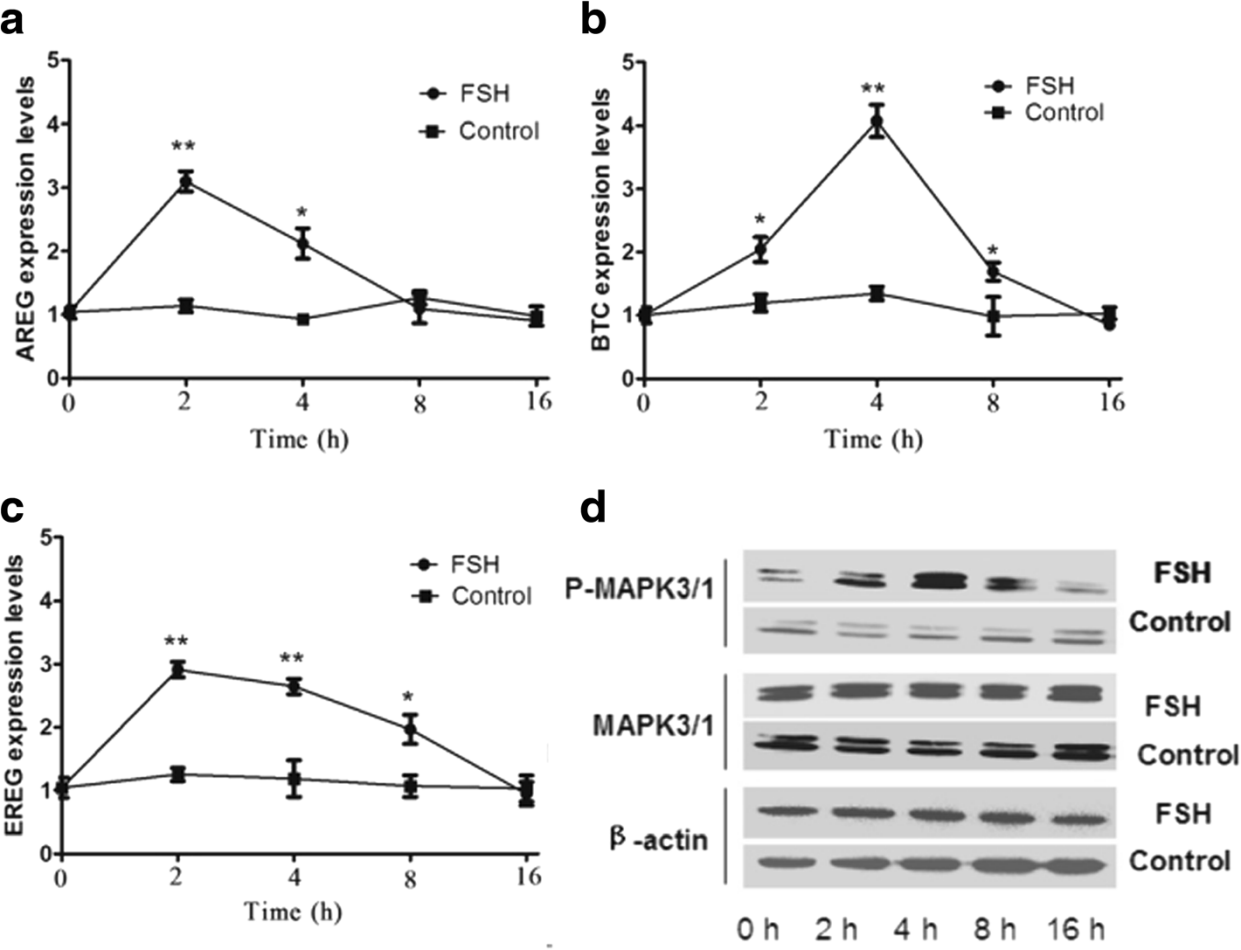
The effects of FSH on the mRNA expression of EGF-like factors and the activation of MAPK3/1 in COCs. **a** The mRNA expression of AREG after stimulation with or without FSH at different times. **b** The mRNA expression of BTC after stimulation with or without FSH at different times. **c** The mRNA expression of EREG after stimulation with or without FSH at different times. **d** The activation of MAPK3/1 after stimulation with or without FSH at different times. The results are presented as the mean ± SEM and are representative of three independent experiments. *P < 0.05 and **P < 0.01 compared with each corresponding group in the control

Previous reports showed that the release of EGF-like factors [31, 32] and the activation of MAPK3/1 [33] are necessary for FSH-induced oocyte meiotic resumption. We examined the effect of FSH on the expression of EGF-like factors mRNA and MAPK3/1 activity after 0 h, 2 h, 4 h, 8 h and 16 h of culture, respectively. FSH significantly stimulates the mRNA expression of EGF-like factors, including amphiregulin (AREG), betacellulin (BTC) and epiregulin (EREG), at 2 h (Fig. 2). The expression of AREG, BTC and EREG transcripts increased within 2 h of FSH stimulation. AREG and EREG reached peak levels by 2 h, but BTC did not. The expression of all three transcripts decreased by 8 h. In the control groups without FSH treatment, low levels of active forms of MAPK3/1 were detected in COCs. In the FSH-induced group, MAPK3/1 activation was clearly detected in COCs at 2–8 h, but did not at 16 h, and the greatest activation was observed at 4 h (Fig. 2d).

### EGF induces oocyte meiotic resumption, NPR2 mRNA decrease and MAPK3/1 activation

**Fig. 3:**
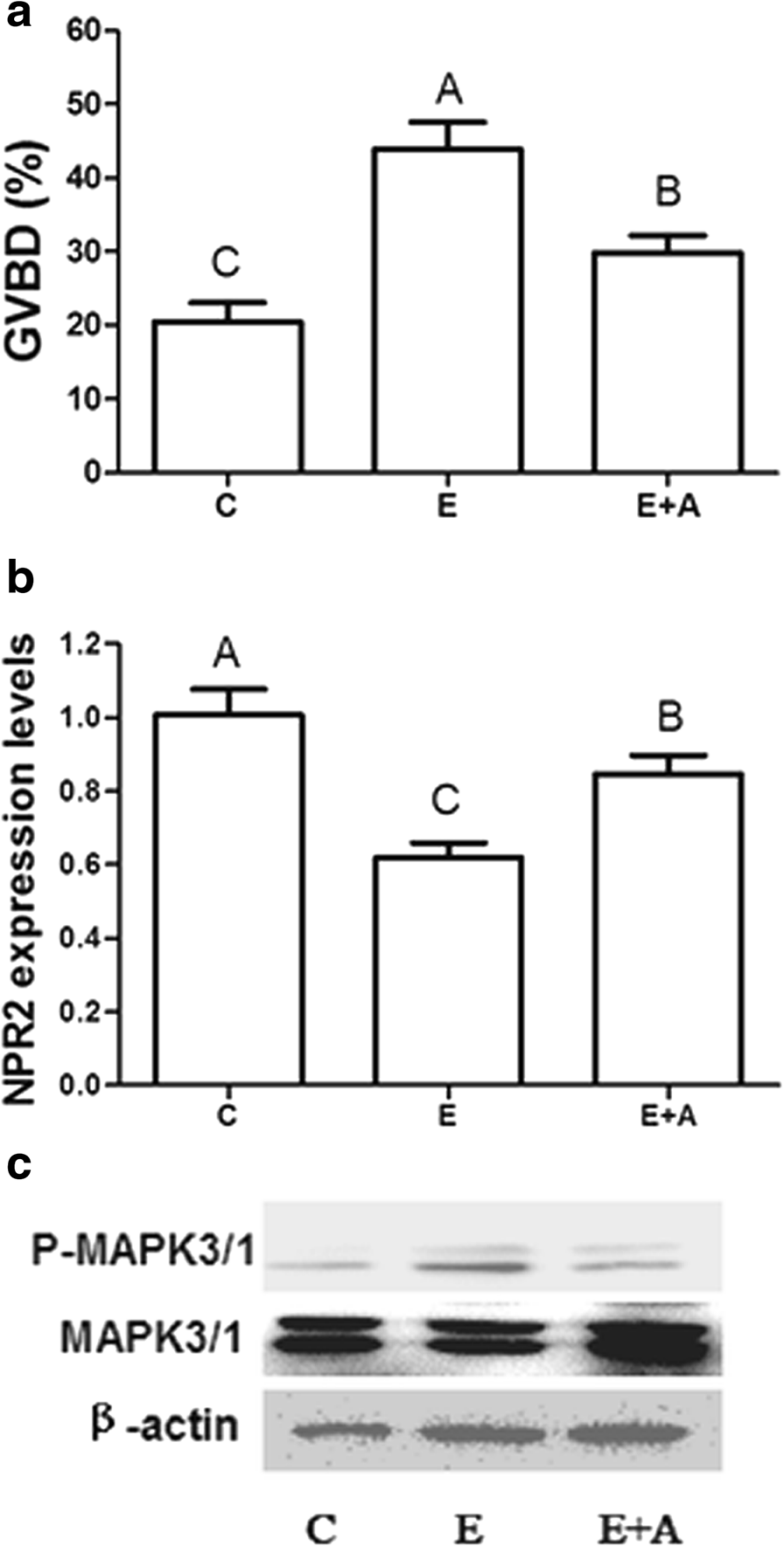
The effects of EGF on oocyte meiotic resumption, NPR2 mRNA expression and MAPK3/1 activation in COCs at 4 h. **a** The GVBD rates after stimulation with EGF or EGF plus AG1478 in COCs. **b** The mRNA expression of NPR2 after stimulation with EGF or EGF plus AG1478 in COCs. **c** The phosphorylation of MAPK3/1 after stimulation with EGF or EGF plus AG1478 in COCs. The results are presented as the mean ± SEM and are representative of three independent experiments. C: Control; E: EGF; A: AG1478. Different letters represent significant differences (*P* < 0.05)

To investigate the requirement for EGFR activity in EGF-induced GVBD, the EGFR inhibitor AG1478 was added to the medium along with 100 ng/ml EGF cultured for 4 h. The results showed that 10 μM EGFR inhibitor AG1478 prevented the oocyte meiotic resumption induced by 100 ng/ml EGF (Fig. 3a). At the same time, AG1478 partly reversed the EGF-induced decrease of NPR2 mRNA levels in COCs (Fig. 3b) and blocked the activity of MAPK3/1 in the presence of EGF (Fig. 3c).

### U0126 inhibits FSH and EGF-induced oocyte meiotic resumption, NPR2 mRNA decrease and MAPK3/1 activation

**Fig. 4:**
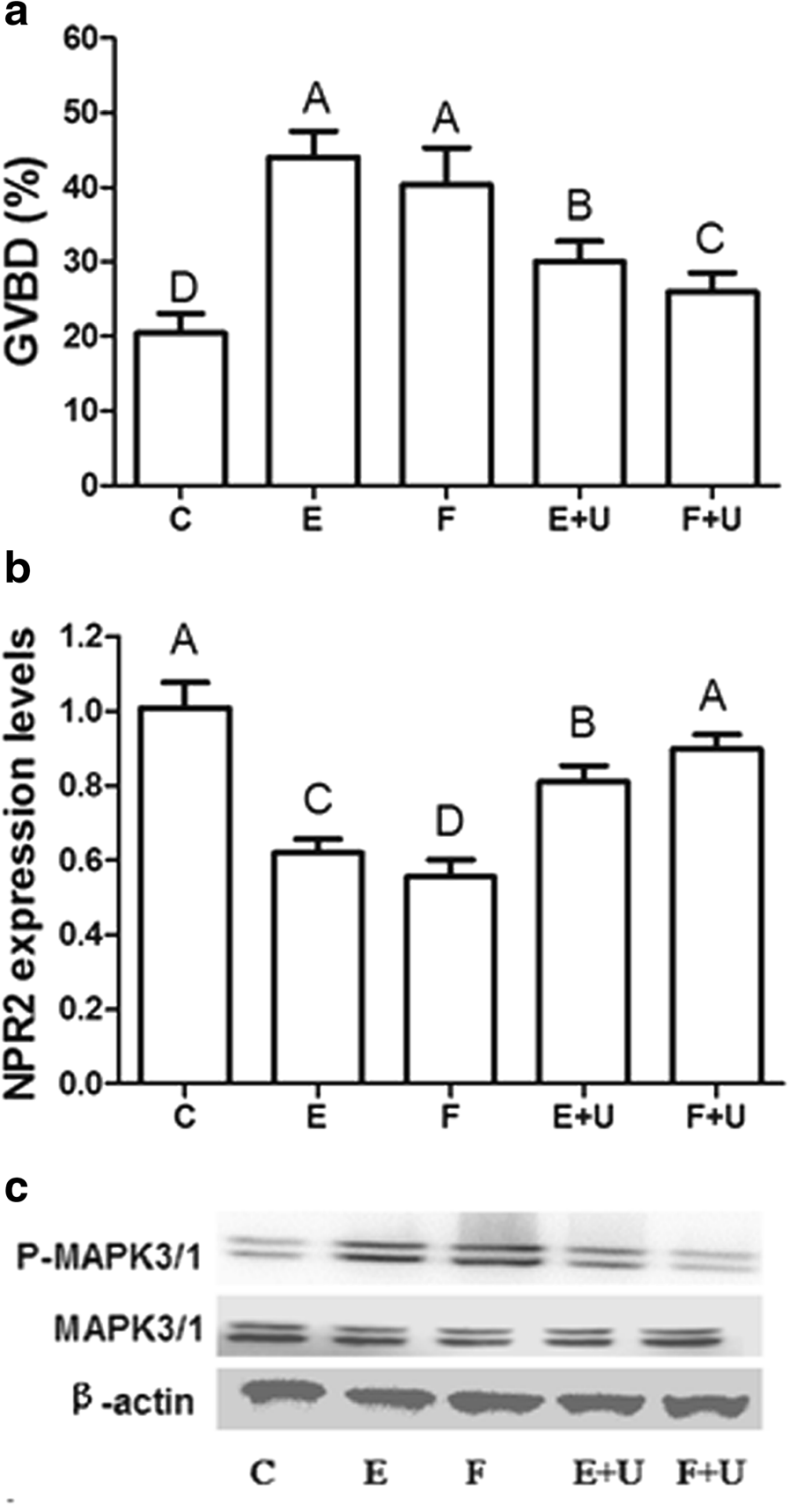
The effects of U0126 on FSH- and EGF-induced oocyte meiotic resumption and NPR2 mRNA expression in COCs at 4 h. **a** The GVBD rates after stimulation with FSH, EGF, FSH plus U0126 or EGF plus U0126 in COCs. **b** The mRNA expression of NPR2 after stimulation with FSH, EGF, FSH plus U0126 or EGF plus U0126 in COCs. **c** The phosphorylation of MAPK3/1 after stimulation with FSH, EGF, FSH plus U0126 or EGF plus U0126 in COCs. The results are presented as the mean ± SEM and are representative of three independent experiments. C: Control; E: EGF; U: U0126; F: FSH. Different letters represent significant differences (*P* < 0.05)

Whether MAPK3/1 is involved in the FSH-induced decrease in NPR2 mRNA and the mechanism by which this is regulated has never been studied. When mouse COCs were cultured and treated with EGF or FSH for 4 h, MAPK3/1 was activated and GVBD occurred (Fig. 4). In addition, 5 μM U0126 clearly inhibited the EGF- or FSH-stimulated oocyte meiotic resumption and MAPK3/1 activation (Fig. 4a and c). Furthermore, U0126 partly inhibited the EGF- and FSH-induced decrease in NPR2 (Fig. 4b).

### EGFR is involved in FSH-induced oocyte meiotic resumption, NPR2 mRNA decrease and MAPK3/1 activation

**Fig. 5:**
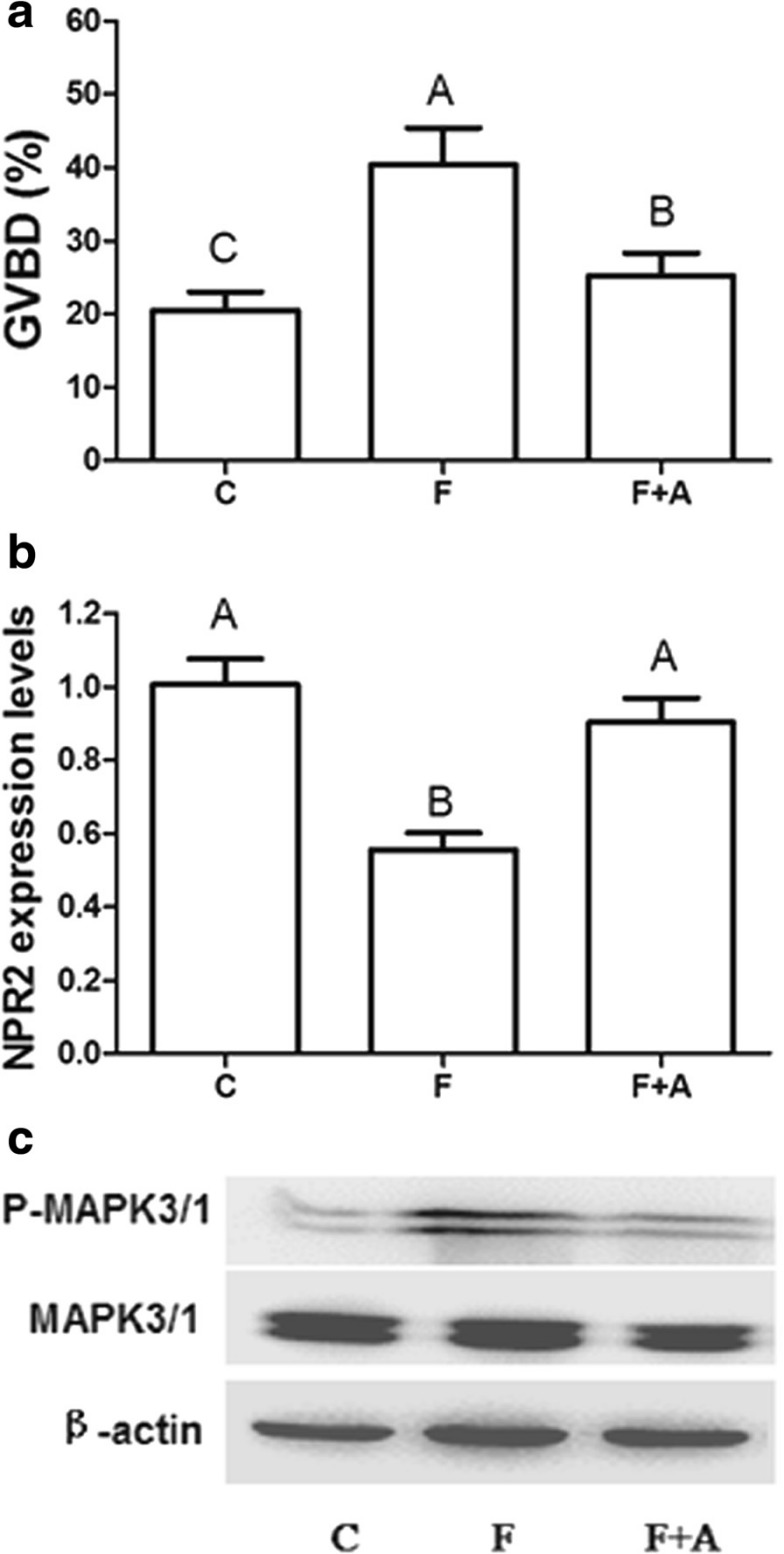
The effects of AG1478 on FSH-induced oocyte meiotic resumption, NPR2 mRNA expression and MAPK3/1 activation in COCs after culture for 4 h. **a** The GVBD rates after stimulation with FSH or FSH plus AG1478 in COCs. **b** The mRNA expression of NPR2 after stimulation with FSH or FSH plus AG1478 in COCs. **c** The phosphorylation of MAPK3/1 after stimulation with FSH or FSH plus AG1478 in COCs. The results are presented as the mean ± SEM and are representative of three independent experiments. C: Control; E: EGF; A: AG1478; F: FSH. Different letters represent significant differences (*P* < 0.05)

Because both FSH and EGF stimulated oocyte meiotic resumption, a decrease in NPR2 levels and MAPK3/1 activation, we designed the following experiment to determine whether the induction of FSH on oocyte meiotic resumption, NPR2 mRNA decrease and MAPK3/1 phosphorylation is mediated by EGFR. FSH was added to the medium containing 10 μM AG1478, and the FSH-induced oocyte meiotic resumption was decreased by the presence of AG1478 after culture for 4 h (Fig. 5a). In agreement with its effect on FSH-induced oocyte meiotic resumption, AG1478 also inhibited the FSH-induced MAPK3/1 activation (Fig. 5c). In addition, the FSH-induced decrease in NPR2 was partly inhibited by AG1478 (Fig. 5b).

## Discussion

A large number of studies have addressed the mechanism of FSH-induced oocyte maturation; however, the mechanism of oocyte maturation remains unclear. Recent studies suggested that CNP/NPR2 signaling acting as an oocyte maturation inhibitor (OMI) existed in the follicle fluid and maintained meiotic arrest [2]. Therefore, we first detected the pattern of NPR2 expression in COCs cultured in vitro after stimulation with FSH. The data from our study showed that NPR2 mRNA was significantly decreased during in vitro culture with FSH. Moreover, the downregulation of NPR2 mRNA in the COCs occurred between 4 h and 8 h of culture compared with the control group, and approximately 50 % of oocyte germinal vesicle breakdown occurred during this period. These results further support the possible role of this gene in the regulation of oocyte meiotic resumption. Our study reported that FSH decreased the expression of NPR2 in the cultured COCs of rodent species, which was in agreement with a recent study showing that the expression of NPR2 significantly decreased in FSH-stimulated porcine COCs cultured in vitro [26]. However, in vivo studies found that FSH induced CNP and NPR2 expression [34–36] in mouse and rat follicles. The reasons for the different in vitro and in vivo results may be because FSH stimulates follicle development in vivo [37], and bigger follicles contain higher expression levels of NPR2 [2, 38].

Previous studies have indicated that the gonadotropin-responsive ovarian paracrine pathway modulates gene expression. This pathway involves LH-dependent intraovarian expression of EGF-like factors that are processed and released from the cell surface to activate EGFR in a paracrine fashion, further leading to the activation of the MAPK3/1 cascade, which regulates various cellular processes through activation of additional kinases or transcription factors to modulate gene expression [39, 40]. In addition, there are also reports that EGFR [27] and MAPK3/1 [41, 42] are involved in oocyte meiotic resumption. So we tested the involvement of MAPK3/1 and EGFR in FSH-induced oocyte meiotic resumption. The results showed that FSH stimulated the expression of EGF-like factors and the phosphorylation of MAPK3/1 in COCs. Our result is consistent with previous reports that FSH stimulated the expression of EGF-like factors [31, 32] and activated MAPK3/1 [33]. Further study revealed that EGF induced oocyte meiotic resumption, NPR2 mRNA decrease and MAPK3/1 activation. Additionally, EGF-mediated NPR2 mRNA decrease and meiotic resumption could be reversed with the EGFR inhibitor AG1478. This is similar to the results of a previous study that showed that the activation of EGFR by EGF suppressed NPR2 mRNA levels and meiotic resumption in mouse COCs [27]. FSH [39, 40] and EGFR [43] signaling-activated MAPK3/1 were associated with oocyte meiotic resumption [41, 42] and NPR2 mRNA decrease [27, 44]. In our study, we also found that blocking of MAPK3/1 activity by U0126 partly inhibited EGF- and FSH-induced GVBD and NPR2 mRNA decrease. Our results were similar to a previous report that the inhibition of MAPK3/1 activity by U0126 partly inhibited EGF-induced meiotic resumption [27].

## Conclusions

We provide the first report that NPR2 is involved in FSH-mediated mouse oocyte meiotic resumption and that this process depends on the EGFR and MAPK3/1 signaling pathways. These results offer promising insights for identifying new components of the signaling pathways that may be involved in FSH-stimulated oocyte meiotic resumption.

## Abbreviations

AREG: amphiregulin
BTC: betacellulin
cAMP: cyclic adenosine monophosphate
CDK1: cyclin dependent kinase 1
cGMP: cyclic guanosine monophosphate
CNP: C type natriuretic peptide
COCs: cumulus oocyte complexes
EGF: epidermal growth factor
EREG: epiregulin
FSH: follicle stimulating hormone
GVBD: germinal vesicle breakdown
LH: luteinizing hormone
MAPK: mitogen activated protein kinase
MI: metaphase I
MII: metaphase II
MPF: maturation promoting factor
NPR2: natriuretic peptide receptor 2
OMI: oocyte maturation inhibitor
PDE3A: phosphodiesterase 3A
